# Penetration of different molecular weight hydrolysed keratins into hair fibres and their effects on the physical properties of textured hair

**DOI:** 10.1111/ics.12663

**Published:** 2020-10-15

**Authors:** E. Malinauskyte, R. Shrestha, P. A. Cornwell, S. Gourion‐Arsiquaud, M. Hindley

**Affiliations:** ^1^ TRI Princeton 601 Prospect Avenue Princeton NJ 08540 USA; ^2^ Croda Europe Ltd Foundry Lane Widnes WA8 8UB UK

**Keywords:** hair treatment, hydrolysed protein, keratin, penetration, peptide, tensile properties, textured hair

## Abstract

**Objective:**

To investigate the effects of different molecular weight (MW), wool derived hydrolysed keratins (i.e. peptides) on the physical properties of relaxed textured hair.

**Methods:**

Very curly hair of African origin was relaxed using sodium hydroxide‐based treatment. Relaxed hair was treated with different MW peptides derived from keratin protein and an amino acid, L‐Leucine. The low‐MW keratin peptides were 221 Da, the mid‐MW keratin peptides were approximately 2577 Da, and the high‐MW keratin peptides were approximately 75 440 Da. The penetration of these different peptides into relaxed hair was evaluated using a laser scanning micrometre and by fluorescence microscopy. The effect of these compounds on single‐fibre mechanical properties and thermal properties was evaluated using tensile and DSC testing, respectively.

**Results:**

Low‐ and mid‐MW compounds were able to penetrate deep into the hair cortex. High‐MW peptide adsorbed onto the hair surface and possibly slightly penetrated into the outer layers of the fibre surface. Both mid‐ and high‐MW keratin peptides, increased Young’s modulus and reduced hair breakage at 20% and 80% relative humidity. With the exception of mid‐MW peptide, other peptides and amino acid were not able to modify thermal properties of relaxed textured hair.

**Conclusions:**

Our data suggest that low‐MW compounds may increase hair volume, and high‐MW peptides may repair damage on freshly relaxed textured hair.

## Resumé

**Objectif:**

Etudier les effets des kératines hydrolysées, issues de la laine, de différents poids moléculaires (PM) (par ex : peptides) sur les propriétés physiques des cheveux texturés défrisés.

**Méthodes:**

Les cheveux très bouclés d’origine africaine ont été défrisés à l’aide d’un traitement à base d’hydroxyde de sodium. Les cheveux défrisés ont été traités avec des peptides de différents PM dérivés de la protéine de kératine et un acide aminé, la L‐leucine. Les peptides de kératine de PM faible étaient de 221 Da, les peptides de kératine de PM moyen étaient d’environ 2 577 Da et les peptides de kératine de PM élevé étaient d’environ 75 440 Da. La pénétration de ces différents peptides dans les cheveux défrisés a été évaluée à l’aide d’un micromètre à balayage laser et par microscopie à fluorescence. L’effet de ces composés sur les propriétés mécaniques à fibre unique et les propriétés thermiques a été évalué à l’aide de tests de traction et de l’analyse calorimétrique différentielle (ACD), respectivement.

**Résultats:**

Les composés de PM faible et moyen ont pu pénétrer en profondeur dans le cortex des cheveux. Les peptides de PM élevé ont été adsorbés sur la surface des cheveux et ont éventuellement pénétré, légèrement, dans les couches externes de la surface des fibres. Les peptides de kératine de PM moyen et élevé ont augmenté le module de Young et réduit la casse des cheveux à 20 % et 80 % d’humidité relative. À l’exception des peptides de PM moyen, d’autres peptides et acides aminés n’ont pas pu modifier les propriétés thermiques des cheveux texturés défrisés.

**Conclusions:**

Nos données suggèrent que les composés de PM faible peuvent augmenter le volume des cheveux et que les peptides de PM élevé peuvent réparer les dommages sur les cheveux texturés fraîchement défrisés.

## Introduction

Human hair is a unique bio‐substrate, ideally adapted to fulfil its function as a protective and insulating shield for our skin. Hair fibres consist of an inner cortex, encased in a protective cuticle layer, which makes up most of the hair mass and provides much of hair’s mechanical strength as well. The key structural proteins in the hair cortex are (a) the keratins that constitute the intermediate filaments, and (b) keratin‐associated proteins that form the matrix surrounding intermediate filaments. Both protein types are responsible for the hair’s tensile strength. Matrix proteins help provide flexibility to the hair as well.

Although hair is naturally a very flexible and strong material, consumers choose to chemically modify, wash, treat and style their hair to change their appearance and look their best. Unfortunately, these practices can induce severe alterations in the hair. Furthermore, hair condition is made worse daily through exposure to chlorine from swimming pool water and environmental factors such as sunlight and pollution. To address these problems, a multitude of materials are available for the cosmetic formulator to develop various cosmetic treatments. Several approaches were designed to restore damaged hair condition, including hair lipid repair, cuticle surface repair and internal hair protein repair. Internal protein repair technologies that recently piqued interest in the industry include carboxylic acids [1, 2, 3], divalent metal‐carboxylic acid salts [4, 5], specially designed hydrogen bond builders [6] and cross‐linkers [7, 8]. However, one of the most widely used classes of hair protein repair agents in the industry is hydrolysed proteins (peptides). Wool‐derived keratin peptides are particularly popular. The use of proteins to repair damage in hair protein has substantial consumer appeal. Several studies have investigated the hair benefits provided by peptides. Gamez‐Garcia (1993) showed that treatment with hydrolysed wheat proteins and wheat oligosaccharides modified the stress relaxation of virgin (undamaged) hair at high and low humidities [9]. Barba *et al*. [10, 11, 12] showed that wool keratin peptides can restore the surface properties, moisture content, tensile properties and water adsorption of bleach‐damaged hair. Benaiges *et al*. [13] claimed that protein‐rich plant extracts can improve the mechanical properties of bleach‐treated hair. Cruz *et al*. [14] have performed a very wide‐ranging screening of 10–13 amino acid synthetic peptides and determined those peptides that bind well to hair keratins. Subsequent work showed hair benefits such as straightening for curly hair and volume for straight hair [15, 16].

Although the effect of proteins on hair fibres has been well studied over recent decades, the majority of studies have looked at protein effects on straight or wavy hair and on repair from bleaching or semi‐permanent colorant‐related damage. Little research has been performed with proteins on textured hair, or textured hair that has been chemically straightened.

Textured hair fibres have a unique, highly curled and coiled structure that causes increased tangling and grooming damage. The structure of these fibres also makes them more prone to fracture when stretched [17, 18]. Many consumers with textured hair use chemical treatments, including relaxers and perms, to straighten their hair. These treatments damage hair proteins, making the hair even more fragile [19]. Clearly, there is a consumer need for hair care products that can, somehow, restore these damaged hairs.

This study investigated the effects of a series of wool‐derived hydrolysed keratins on very curly hair that had been straightened with a sodium hydroxide‐based relaxer treatment. There are relaxers available that do not contain sodium hydroxide (no‐lye relaxers which use lithium hydroxide and guanidine carbonate); however, we selected the relaxer that is popular in the region where the hair came from. The hydrolysed keratins varied in average MW and comprised of the following: (i) a low‐MW hydrolysed keratin (221 Da), (ii) a mid‐range MW hydrolysed keratin (2577 Da) and (iii) a high‐MW hydrolysed keratin (75440 Da). Leucine (131 Da) was used as an amino acid reference treatment. Leucine and the respective hydrolysed keratins’ effects on the dimensional, mechanical and thermal properties of the hair were investigated using hair diameter measurements, single‐fibre tensile and differential scanning calorimetry (DSC), respectively. Additionally, the penetration of the mid‐range and high‐MW peptides into the relaxed textured hair was investigated using fluorescence microscopy.

## Materials and methods

### Hair samples

Single‐source hair was collected from South African volunteers who had not previously used any chemical treatments. The L’Oréal classification [20] was used to assess hair curl type. The hair was assessed to have type VII curl.

### Relaxing procedure

Hair was gathered into 0.5 g bundles. Each bundle was washed first with 15% w/v sodium lauryl ether sulphate solution in deionized (DI) water (pH 5.2) and dried at ambient conditions. Then, 5 g of relaxer (Sofn’ Free Crème Relaxer, M & M, South Africa) was applied to 0.5 g hair, combed through and left for 15 min, while massaging every 5 min. Hair was then rinsed for 2 min under a tap with a fixed water temperature and flow rate (Intellifaucet^™^ tap by Hass Manufacturing Company, Averill Park, NY; 40°C, flow rate 3.8 L min^−1^). A total of 0.2 mL of pH 6 neutralizing shampoo (Softn’ Free, M & M, South Africa) was applied per 1 g hair, and massaged for 30 s, and rinsed for 30 s under a tap (40°C, flow rate 3.8 L min^−1^). The washing process was repeated one additional time, and the hair was left to dry under ambient conditions.

### Amino acid and keratin peptide ingredients

L‐Leucine (≥98% purity) was obtained from Sigma‐Aldrich, (MO, USA). Aqueous solutions of low‐MW (221 Da) hydrolysed keratin (INCI: hydrolysed keratin), mid‐range MW (2577 Da) hydrolysed keratin (INCI: Aqua (and) hydrolysed keratin) and high‐MW (75 440 Da) hydrolysed keratin (INCI: Aqua (and) keratin (and) hydrolysed keratin), respectively, were supplied by Croda (UK).

### Preparation of fluorescently labelled keratin peptides

An amine reactive label, 5(6)‐carboxytetramethylrhodamine N‐succinimidyl ester (TMR‐SE, Sigma‐Aldrich, USA), was used for labelling the peptides, henceforth referred to as the label. Before labelling, hydrolysed keratin solutions were dialysed against phosphate‐buffered saline (PBS) using a dialysis cassette (Slide‐a‐lyzer, Thermo Scientific, USA) with a MW cut‐off of 2000 Da to remove any amine‐containing substances from the products. Hydrolysed keratin concentrations in dialysed solutions were determined using BCA protein assay (Pierce™ BCA Protein Assay Kit, Thermo Scientific, USA) per manufacturer instructions. A total of 1 mg of the label was used per 10 mg peptide.

For label‐only control, the same amount of label was added to a sodium bicarbonate buffer without any peptides in it. The reaction was incubated for 1 h at room temperature with continuous stirring. The labelled peptides were further purified by dialysis or centrifugal filter unit (Amicon Ultra‐15 Centrifugal Filter Units, Millipore Sigma, USA) to remove any unbound free label. Labelled peptides were used for hair soaking treatments to determine penetration.

### Penetration of labelled keratin peptides into hair fibres

Penetration studies were performed on relaxed textured hair. People with this hair type tend to use long‐duration treatments, including those designed for overnight use (while wearing a cap). To understand penetration behaviour, three‐hour soaking was selected for this study. A total of 10 mg of hair (~20–30 × 1‐inch hair fibres) was soaked in 1.5 mL labelled keratin peptide solution such that hair fibres were fully immersed in the solution. Hair samples were incubated for 3 h with gentle rocking. After incubation, hair was rinsed in DI water for 1 min and air‐dried on a paper towel. Hair fibres were embedded as a bundle in OCT compound (Optimal cutting temperature, Sakura Finetek USA, Inc, Torrance, CA, USA) prior to cryosectioning. Several hair cross‐sections (5 µm) were collected for image analysis.

Hair cross‐section images were collected on a Leica DM8 (Leica biosystems, Wetzlar, Germany), equipped with the Leica DFC3000 G camera. Images were acquired using the Leica Application suite X (LAS X) software. Each image was recorded using two separate channels: green (Excitation:460–500 nm/DC:505 nm/Emission:512–542 nm), exposure 150 ms, and red (Excitation:532–558 nm/DC:565 nm/Emission:570–640 nm), exposure 2 ms.

### Scanning electron microscopy

Scanning electron microscopy (SEM) images were obtained using a high‐resolution field emission scanning electron microscope FEI XL30 FEG‐SEM (Philips, The Netherlands). Random fibres were chosen from selected hair bundles. A total of 15 fibres from each control group were studied. Fibres were divided in half and fixed on a stud to assure approximately 5 cm hair length analysis. The hair fibres were attached to the surface of a 2.5 cm diameter stainless steel disc using double sided carbon tape and coated with Iridium to approximately 7 nm. The SEM pictures were recorded at 10 KV accelerating voltage with a working distance of ~10 mm.

### Hair diameter and single‐fibre tensile testing studies

Relaxed hair bundles were soaked in 1% w/v aqueous solutions of L‐Leucine, low‐MW, mid‐ MW and high‐MW keratin peptides for 24 h while constantly shaking (HY‐5 orbital shaker, Zenith Lab Inc., CA, USA). Both relaxed and virgin hair controls were soaked in DI water for 24 h while shaking as well. It was thought that this duration, although prolonged, but still relevant to the hair type, would show the changes in mechanical hair property better. After 24 h, all hair samples were rinsed with DI water for 30 s and air‐dried at 20 ± 2°C and 60% relative humidity (RH).

Fibres were mounted in brass crimps, and dimension measurements were performed at 20 ± 2°C and 60% RH. The maximum and minimum diameters of the crimped fibres (150 per group) were measured at 5 locations using a laser scanning micrometre (FDAS770 Fibre Dimensional Analysis System, Diastron Ltd, UK). Cross‐sectional area was calculated using UVWin 3.6 build 3 software (Diastron Ltd, UK). Stress‐strain curves of fibres were recorded at 20% and 80% RH (≥15 h equilibration after water evaporated from the wet state) using a MTT690 Miniature Tensile Tester (Diastron Ltd, UK). A total of 50 fibres from each group were extended at 40 mm min^−1^ rate. Young’s modulus was calculated by analysing the slope in the 0.2–1% extension region to ensure that the analysis was done in the linear region.

### Differential scanning calorimetry

Thermal properties of hair were tested using DSC2500 instrument (TA Instruments, DE, USA). Five replicates were prepared for each sample. For this study, the same hair and equilibration conditions were used as for the fibre dimension study. Approximately 10 mg of finely chopped hair was placed in a high‐pressure hermetic DSC pan together with 50 µL of DI water. The heating rate was 5°C min^−1^. Prior to testing, the instrument was calibrated using an indium standard.

### Statistical analysis

Pearson's chi‐squared test was used to evaluate whether the observed differences in premature breakage are dependent or independent of treatments. Statistical differences of all results were analysed using JMP™ 14.0.0 analytical software (SAS Institute Inc., NC, USA). Statistically significant differences were evaluated using a Dunnett’s test at 95% confidence level via comparing each treatment group to (a) relaxed hair treated with DI water (i.e. relaxed hair control) and (b) virgin hair treated with DI water (i.e. virgin hair control). The outliers were removed using the Tukey quartile method (includes median). Graphs are presented as means ± the respective standard errors of mean.

## Results

### Effects of peptide treatments on fibre cross‐sectional area

**Figure 1:**
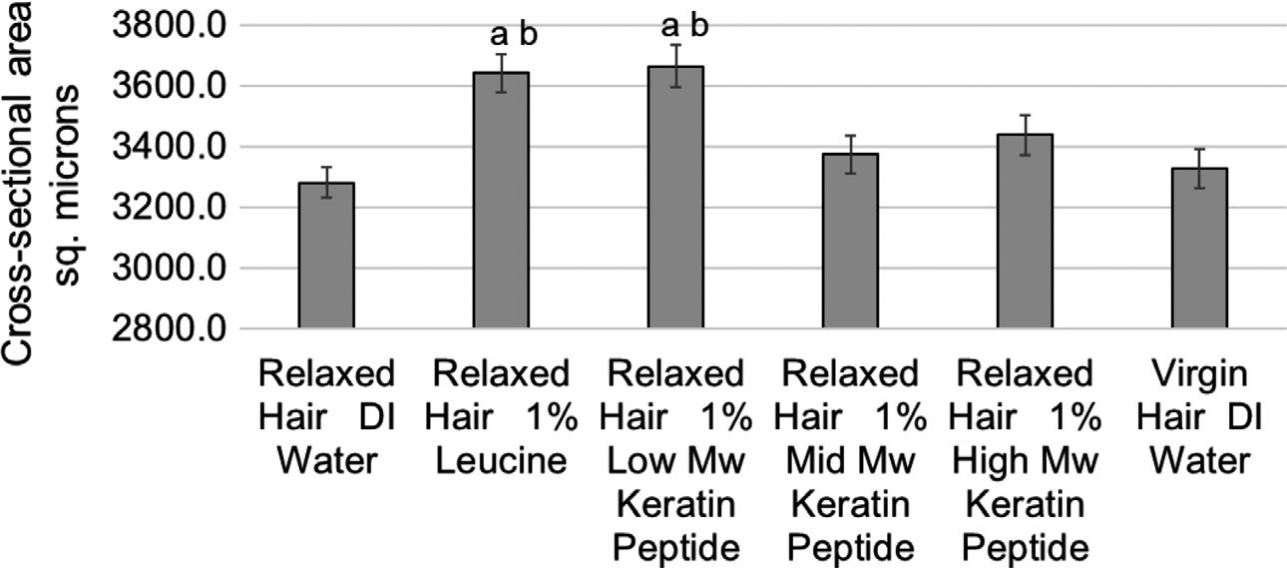
Cross‐sectional area of fibres as a function of treatment and hair condition (means +/‐ standard errors). Results that are statistically different in comparison with relaxed hair control (DI water) are labelled with the letter a, and results that are significantly different from virgin hair control (DI water) are labelled with the letter b.

The increase in fibre cross‐sectional area at ambient humidity, or lateral hair swelling, can be used to investigate the effects of hair treatments on internal hair protein structures. In fact, significant swelling provides evidence that treatments have penetrated into the hair fibre. In this study, the cross‐sectional area of 150 fibres from each group was measured as described above. Fig. 1 presents the cross‐sectional area of hair with different treatments as indicated. Statistical analysis showed that cross‐sectional areas of hair treated with 1% low‐MW keratin peptide and 1% leucine‐treated hair were significantly higher than that of both virgin and relaxed hair controls (11.7% and 11.0%, respectively, higher than relaxed hair control). These results suggest that low‐MW actives effectively penetrated into the damaged hair cortex.

As we were not able to evaluate mid‐ and high‐MW peptide penetration using diameter measurements, we used fluorescence microscopy as a more sensitive technique to investigate small differences in these actives penetrating into hair.

### Penetration of labelled keratin peptides into hair fibres

**Figure 2:**
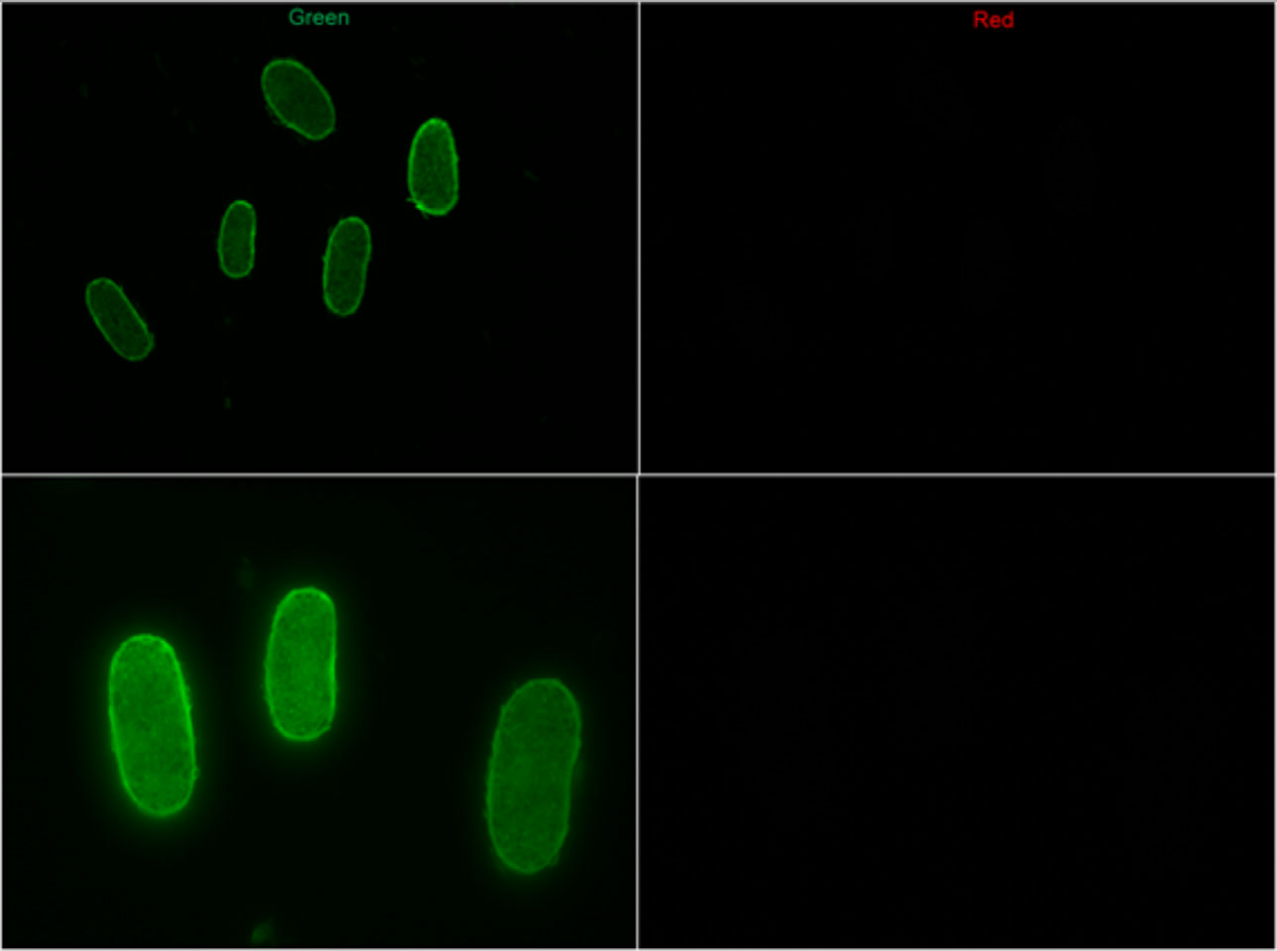
Cross‐sections of Afro‐textured relaxed hair soaked for 3 h in dye solution that was filtered via centrifugal filter to remove free dye at low (20×, top) and high (40×, bottom) magnification.

**Figure 3:**
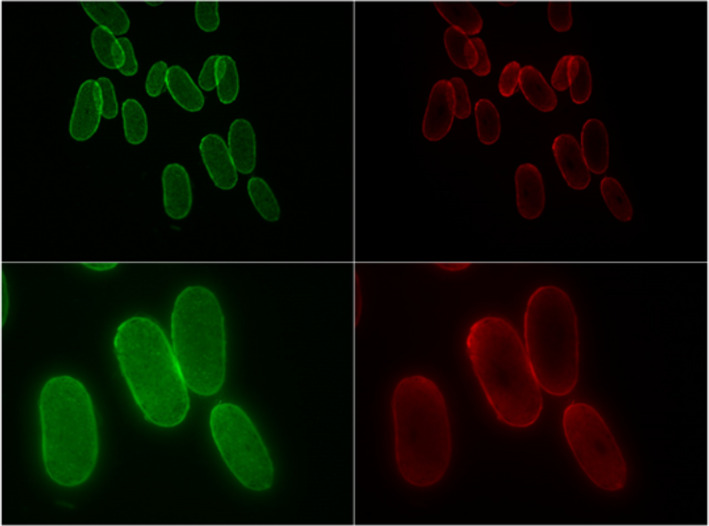
Cross‐sections of Afro‐textured relaxed hair soaked in labelled mid‐MW keratin peptides for 3 h at low (20×, top) and high (40×, bottom) magnification.

**Figure 4:**
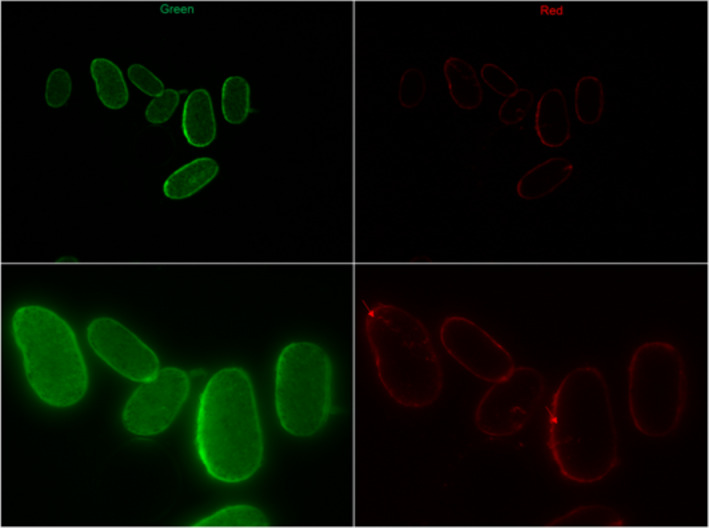
Cross‐sections of Afro‐textured relaxed hair soaked in labelled high‐MW keratin peptides for 3 h at low (20×, top) and high (40×, bottom) magnification.

Figures 2, 3 and 4 show the sections obtained from relaxed control hair and relaxed hair treated with labelled keratin peptides for 3 h. The images taken using the green channel show natural autofluorescence of the hair and help display the positioning of the cross‐sections in the images. The images of the same samples taken using the red channel show only the fluorescence from the labelled hydrolysed keratin. It is interesting to note that, as these were textured hair samples, the cross‐sections were especially flat and elliptical. This is characteristic for this hair type. There was no presence of fluorescence in the red channel with relaxed hair which had been soaked in filtered dye solution for 3 h (Fig. 1), indicating that filtration using the centrifugal filters removes any free dye. Fig. 2 shows that after 3 h of soaking, the mid‐range MW keratin peptides had penetrated deep into the cortex. However, after 3 h soaking, the high‐MW peptide had only just entered the outer layers of the cortex (Fig. 3).

### Pre‐existing mechanical damage of hair via SEM

**Figure 5:**
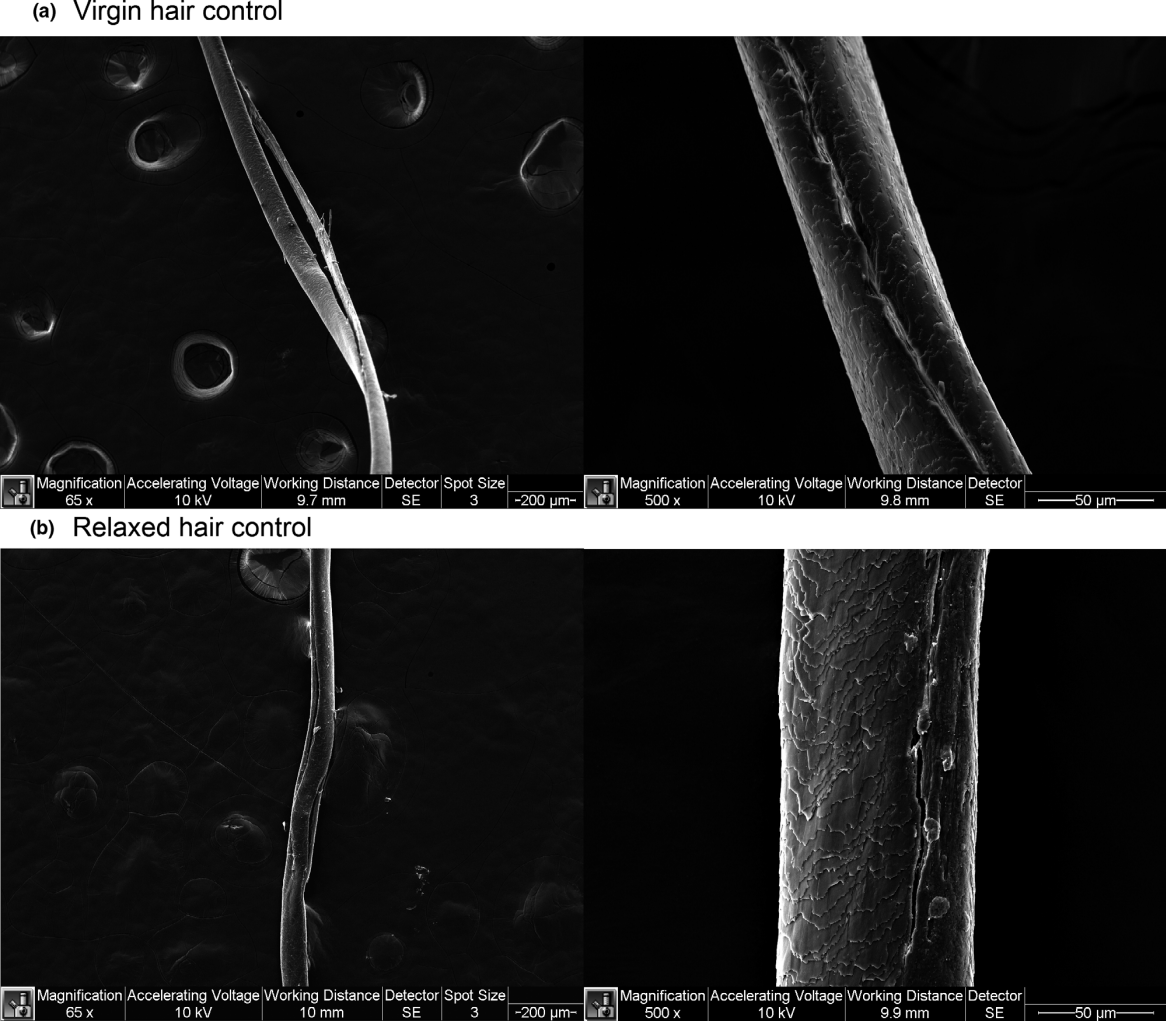
Longitudinal cortex cracks of virgin and relaxed control hair.

Literature suggests that textured hair contains higher levels of mechanical damage as a result of intense grooming regimens [21]. The preliminary evaluation of hair surface condition was performed using SEM. Examples of these cracks in virgin and relaxed hair controls are presented in Fig. 5. All selected fibres were thoroughly investigated from the top of the stud to the bottom. The screening revealed that some fibres contained long longitudinal cracks in certain areas of the cortex: five fibres in virgin group and six fibres in relaxed hair group contained the aforementioned flaws. Some of the fibres had more than one longitudinal crack. Some cracks were widely open (65× magnification), whereas others showed clear signs of cracking; however, the cracks’ depth was unknown (500× magnification). Because of a relatively small sample size (15 fibres per group), we consider these results preliminary and further research is required in order to study the relationship between longitudinal cracks and mechanical properties of hair.

### Surface coverage by mid‐ and high‐MW peptide treatments via SEM

**Figure 6:**
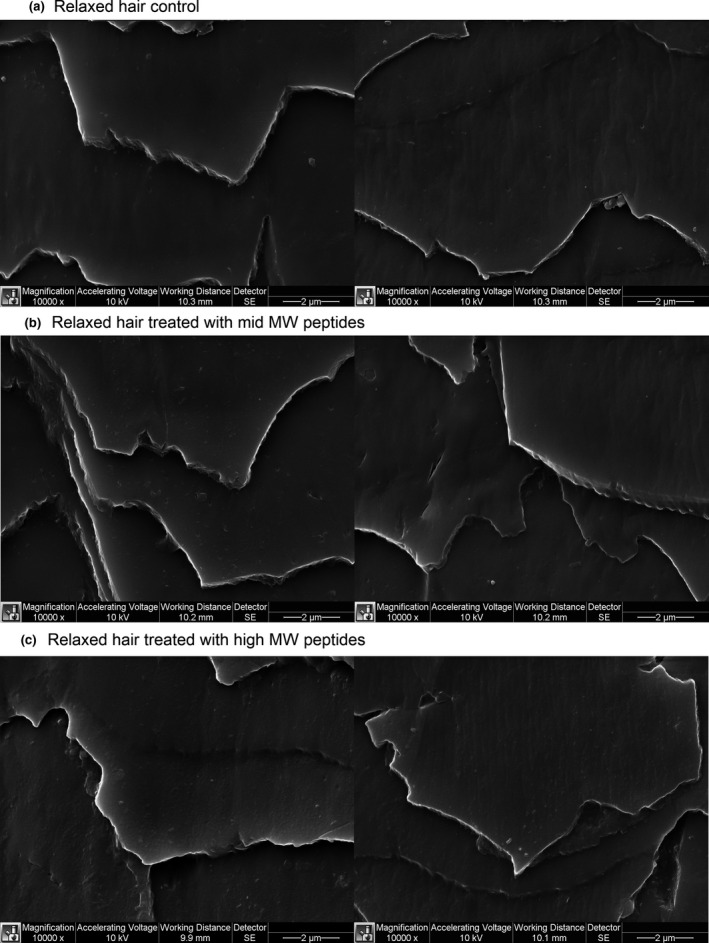
Surface coverage by treatments.

Figure 6 presents SEM images of randomly chosen fibres from the relaxed hair control, mid‐ and high‐MW peptide treatment groups. Only the presence of high‐MW peptide film is visible at 10 000× magnification. This suggests that mid‐MW peptide size is not sufficient to form relatively thick film.

### Effects of peptide treatments on hair stiffness

**Figure 7:**
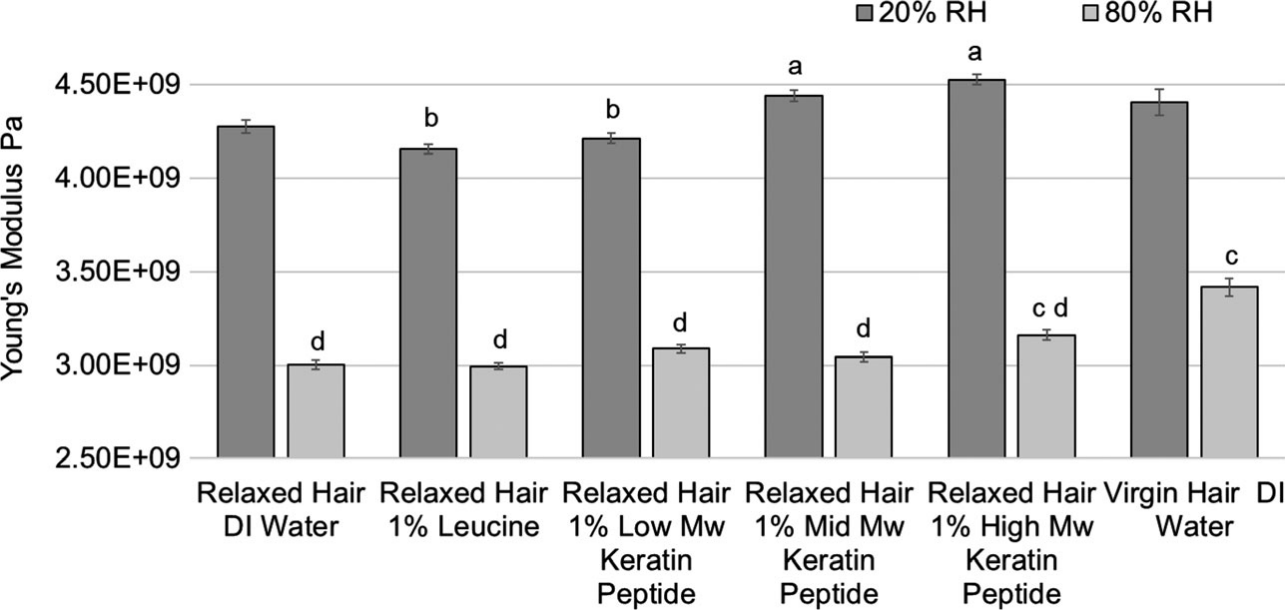
Young’s modulus measured at 20% and 80% RH as a function of treatment and hair condition (means ± standard errors). At 20% RH, results that are statistically different in comparison with relaxed hair control (DI water) are labelled with the letter a, and results that are significantly different from virgin hair control (DI water) are labelled with the letter b. At 80% RH, results that are statistically different in comparison with relaxed hair control (DI water) are labelled with the letter c, and results that are significantly different from virgin hair control (DI water) are labelled with the letter d.

Figure 7 summarizes Young’s moduli data obtained at 20% and 80% RH. Dunnett’s test showed that, at 20% RH, Young’s moduli of hair treated with mid‐ and high‐MW peptides were significantly higher than those of relaxed hair control. Young’s moduli of virgin hair and relaxed hair treated with low‐MW peptide actives were found to be no different than the relaxed hair control. Young’s moduli of low‐MW keratin peptides and leucine‐treated relaxed hair were significantly lower than those of virgin hair control.

An increase in humidity caused a major shift in elastic properties of the hair samples. This was especially visible when comparing virgin with relaxed hair. At 80% RH, Young’s modulus of virgin hair was significantly higher than that of differently treated relaxed hair (14.0% higher than relaxed hair control). When comparing the elasticity of differently treated relaxed hair under humid conditions, only high‐MW peptide treated hair remained significantly stiffer.

### Effects of peptide treatments on hair breakage

The summary of premature breakage (defined as hair breakage at extension ≤ 20% strain) at 20% and 80% RH is presented in Table 1.

**Table 1:** The treatment effect on the premature breakage (number of prematurely broken fibres per 50 tested fibres)

	Relaxed Hair DI Water	Relaxed Hair 1% Leucine	Relaxed Hair 1% Low‐MW Keratin Peptide	Relaxed Hair 1% Mid‐MW Keratin Peptide	Relaxed Hair 1% High‐MW Keratin Peptide	Virgin Hair DI Water	*p* Chi‐Squared Test
20% RH	15	19	15	6	5	12	0.005
80% RH	10	6	9	3	1	4	0.028

At 20% RH, 24% of virgin hair and 30% of relaxed control hair broke prematurely. At 80% RH, the premature breakage in all of the tested hair types was significantly reduced. However, mid‐ and high‐MW peptide contribution to the reduction of premature breakage of relaxed hair at both humidities appears to be stronger than that of other actives. Performed chi‐squared test confirmed that observed breakage trends are associated with the applied treatments (the null hypothesis of the chi‐squared test that no relationship exists between breakage and treatment was rejected).

**Figure 8:**
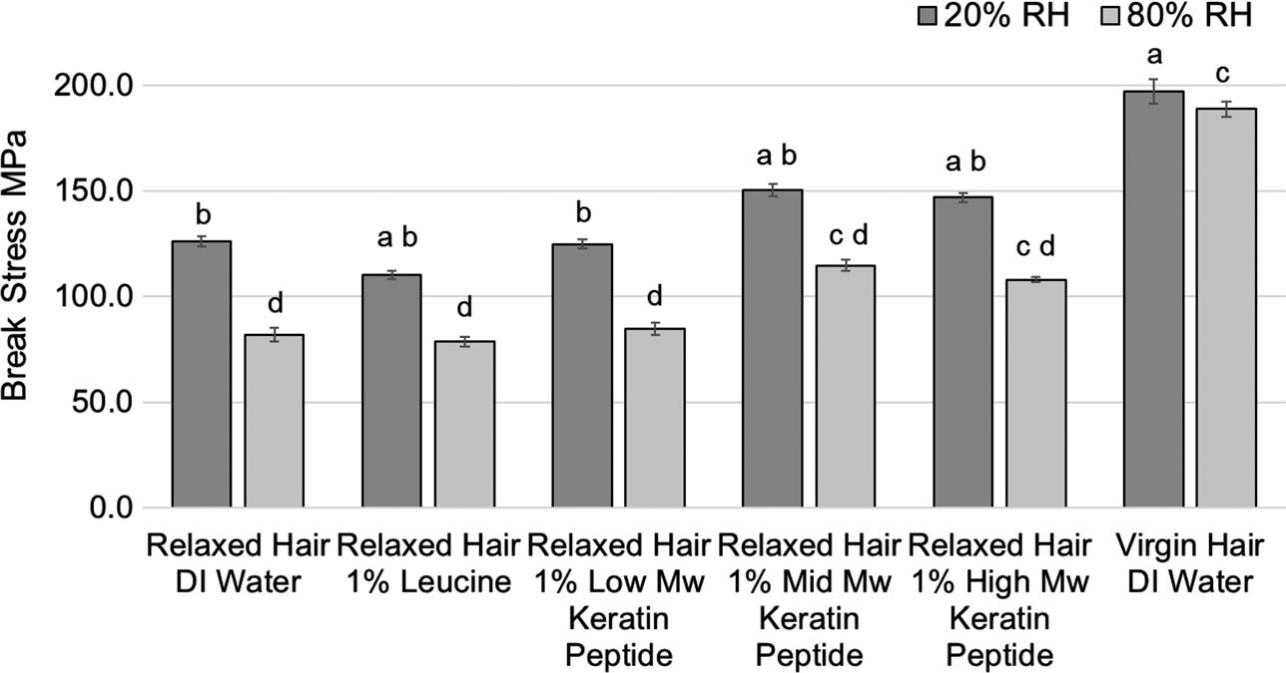
Break stress measured at 20% and 80% RH as a function of treatment and hair condition (means ± standard errors). At 20% RH, results that are statistically different in comparison with relaxed hair control (DI water) are labelled with the letter a, and results that are significantly different from virgin hair control (DI water) are labelled with the letter b. At 80% RH, results that are statistically different in comparison with relaxed hair control (DI water) are labelled with the letter c, and results that are significantly different from virgin hair control (DI water) are labelled with the letter d.

The break stress data at 20% and 80% RH are compared in Fig. 8. As expected, virgin hair was significantly stronger than relaxed hair at both humidities. The difference in virgin hair break stress at both humidities was insignificant. At 20% RH, the break stresses of mid‐ and high‐MW keratin peptides treated hair were significantly higher than that of the relaxed hair control by ~18.6% and ~16.3%, respectively. The break stress of hair treated with leucine was significantly lower than the relaxed hair control.

Whereas we have not found significant difference between virgin hair equilibrated at 20% and 80% RH, the humidity effect on differently treated relaxed hair was significant: break stress drastically decreased with increased humidity. For example, the break stress of relaxed hair control at 80% was lower by 35.3% in comparison with that at 20% RH. Moreover, the higher environmental humidity significantly affected the differences between hair treatments as well. The most notable were the following: break stress of virgin hair, mid‐ and high‐MW peptides treated relaxed hair was higher than that of control relaxed hair by 130.9%, 40.0% and 31.6%, respectively (all statistically significant differences).

### Effects of peptide treatments on thermal properties of hair

**Figure 9:**
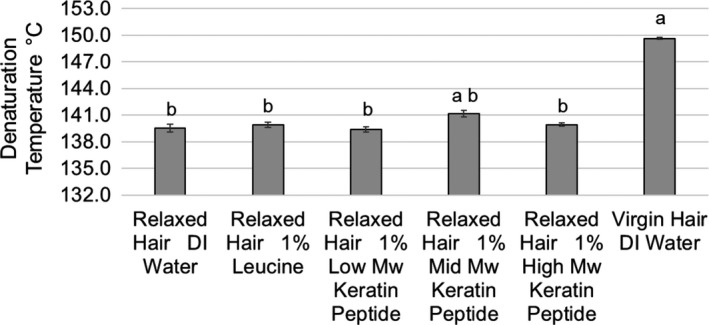
Denaturation temperature as a function of treatment and hair condition (means ± standard errors). Results that are statistically different in comparison with relaxed hair control (DI water) are labelled with the letter a, and results that are significantly different from virgin hair control (DI water) are labelled with the letter b.

After seeing notable changes in mechanical properties, we tested thermal properties of hair expecting to observe significant changes in DSC parameters. The denaturation temperature and enthalpy of virgin hair were 149.7 ± 0.2°C and 19.9 ± 1.3 J g^−1^, respectively. The damaging effect of relaxing treatments was significant: all of the relaxed hair peaks flattened. The dramatic change in the curves shape made it difficult to define the baseline limits which are required to calculate the enthalpies. The denaturation enthalpy of relaxed hair varied between 3 and 5 J g^−1^. The denaturation temperature results are presented in Fig. 9. Statistical analysis did not show a difference in denaturation temperature of differently treated relaxed hair, with the exception of the mid‐MW peptide treated sample. Here, a statistically significant (but relatively small) increase in denaturation temperature (1.7°C) was observed when comparing mid‐MW peptide treated hair with the relaxed hair control.

## Discussion

### Fluorescence microscopy

Fluorescence microscopy techniques, including confocal fluorescence microscopy, have been used for many years for the characterization of hair and for examining the penetration of fluorescently tagged materials [22, 23]. Unfortunately, there is a large amount of natural autofluorescence that can arise from chromophores in the hair, including amino acids with aromatic side chains: tryptophan, tyrosine and phenylalanine. Autofluorescence can also arise from the breakdown products of these amino acids and, of course, melanin [24]. McMullen *et al*. have created a series of excitation–emission matrices for hair fibres and have shown that pigmented hair fibres have two major excitation/emission peaks. One peak that is related to tryptophan has an excitation wavelength at 290 nm and an emission wavelength 335–345 nm (the exact position depending on the level of pigmentation). Furthermore, they report another peak with an excitation wavelength of 366 nm and an emission wavelength of 433 nm; this is related to tryptophan degradation products [25].

Background autofluorescence can be a major problem for penetration studies such as this one, as it can create a false impression of the presence of labelled active in the images. For this reason, the present study used a red fluorescent label that emitted at 579 nm, well above the wavelengths seen with hair autofluorescence. Background autofluorescence was further reduced by looking at treated samples just at 570–640 nm. Despite these efforts, a small amount of background autofluorescence was still observed, and care was taken to take images of control and treated sections with the same microscope and camera settings. Differences in fluorescence were then certainly related to the absorbed peptides.

### Penetration of keratin peptides

Hair fibres have, through evolution, developed a natural barrier to the absorption of chemicals and micro‐organisms called the cell membrane complex (CMC). The CMC runs between all the cells in the hair and comprises two lipid layers sandwiching a protein‐rich layer, known as the δ‐layer [26]. The CMC is the only continuous structure in the hair fibre. Recent studies suggest that the CMC between the cuticle and the cortex contains an additional, highly resistant structure that prevents penetration of materials into the cortex [27]; therefore, many compounds pass very slowly through this barrier, unless the hair is damaged.

The routes of penetration of materials into the hair have been visualized by a number of studies [22, 23, 28]. All these studies show that penetration through the CMC is the main pathway for delivery of actives into the hair cortex. Consistent with these findings, kinetic studies investigating uptake of materials into the hair also show that small molecules penetrate the hair best when they are less ionized, and so, more able to travel through and along the lipid bilayers in the CMC [29]. SEM examination of virgin and relaxed hair revealed an additional pathway that may be common in textured hair: large cracks and pores as a result of pre‐existing mechanical damage (Fig. 5). The penetration to cortex via mechanically damaged surface may be significantly more effective than just sodium hydroxide affected cuticles and CMC.

The results of the dimension measurement study show that only low‐MW actives penetrated into the relaxed hair cortex in large enough quantities to affect hair swelling behaviour. So, despite the damage to the hair caused by the relaxer treatment and the presence of cracks in the hair, only the leucine and low‐MW hydrolysed keratins were able to penetrate deep into the hair in large enough amounts to change protein structures. We propose that although the hair was drying, these compounds, being a charged species, competed with native hair charged side chains over formation of salt‐bridges, as well as contributed to the repulsion of like charged species, which caused the hair cortex to swell by approximately 11%.

Investigation of mid‐ and high‐MW peptide penetration using fluorescence microscopy showed that the mid‐range MW keratin peptide was able to penetrate into the cortex. The high‐MW keratin peptide only penetrated the outer layers of the cortex. We have to conclude that penetration into the hair cortex, despite being visible in the fluorescence microscopy, was not enough from either treatment to affect hair swelling. Although this study provided us a glimpse into the behaviour of peptide penetration, we would like to perform more studies which investigate the kinetics of peptide penetration into the hair in the future.

### Effects of treatments on hair stiffness

Young's modulus measures the resistance of a material to elastic (recoverable) deformation under load. A stiff material has a high Young's modulus and changes its shape only slightly under elastic loads. A flexible material has a low Young's modulus and changes its shape considerably. Young’s modulus of hair changes as a function of relative humidity. At dry conditions, both matrix and intermediate filaments contribute to the hair resistance to stresses. The amorphous matrix, especially of damaged hair, is very sensitive to plasticization by water; therefore, its contribution to hair strength decreases with increasing water content.

Despite significant damage to intermediate filaments (as shown by denaturation enthalpy results), Young’s modulus results suggest that at 20% RH, matrix rigidity masked the damage extent; thus, even severely damaged, relaxed hair was able to resist elastic deformations similarly to virgin hair. There have been suggestions that changes in amino acid composition can occur as a result of the relaxation process [30, 31, 32, 33]. Cleavage of disulphide bond and formation of lanthionine are the most commonly proposed changes [32, 33]. Moreover, studies [30, 31] agree that the levels of negatively charged amino acids remain unchanged. Unfortunately, these data contradict each other, as to whether there is a reduction in lysine [30] or arginine [31] content. Because of these uncertainties, it is entirely reasonable for us to propose that the composition of charged side chains remained the same. Based on this hypothesis, we propose that matrix rigidity of relaxed hair at 20% RH was maintained by (i) the positively and negatively charged side chains (unchanged content suggest similar level of salt‐bridges and hydrogen bonds), and (ii) increased contraction in the matrix due to partial disulphides replacement by lanthionine (shorter chains). This makes the elastic modulus an unsuitable parameter to assess the extent of damage, even between drastically different samples at very dry conditions.

With increasing humidity, differences in Young’s modulus between virgin and differently treated relaxed hair continued to increase, revealing damage to intermediate filaments and to some extent, the matrix. As discussed in the results section, only high‐MW peptide treated hair remained significantly stiffer than the relaxed hair control. Previously published studies investigating effect of peptides on mechanical properties of hair have shortcomings in mechanical testing or its description, such as very low numbers of replicates [10] and unspecified critical testing conditions (such as humidity) [15], making it difficult to make direct comparisons to our results. However, a significant increase in Young’s modulus, which is consistent with our results, was observed after (i) applying mid‐MW peptide (10–12 amino acid) to relaxed hair (tested at 55% RH) [34] (ii) applying γD‐crystallin protein of 21 kDa MW on bleached hair (tested at 62% RH) [35].

### Effects of treatments on hair breakage

Obtained stress‐strain curves shared a common theme – premature breakage. Premature breakage is considered when the hair breaks at extensions below 20% Strain [18]. Kamath et al. postulated that premature breakage may occur because of weak point propagation during tensile experiments. Weak points may be generated by grooming practices in the areas where cross‐section structure changes (fibreflattens or collapses within a short distance in the twist region) [18]. This parameter is highly consumer relevant as real‐life premature breakage occurs under relatively low loads, such as while pulling hair during combing procedures. Performed preliminary SEM screening of several fibres of virgin and relaxed hair controls helped to understand the reason behind such high premature breakage. Fibres contained long longitudinal cracks of various depths. Relaxed hair treated with mid‐ and high‐MW peptides had a fewer number of prematurely broken fibres than other differently treated relaxed hair or virgin hair. It is understandable that fibres with large cracks, like those imaged at 65x magnification, cannot be easily repaired. However, our results suggest that fibres with smaller cracks could have been repaired by mid‐ and high‐MW peptides to mitigate premature breakage. At 80% RH, premature breakage in all of the tested hair types was significantly reduced because of fibre plasticization, especially at stress accumulation points [18]. Nevertheless, mid‐ and high‐MW peptides significantly contributed to the reduction of premature breakage of relaxed hair.

Interesting breakage behaviour was observed while comparing virgin hair at high and low humidities. With other types of hair, it is expected that the average break stress of hair broken at high humidity is lower than that at low humidity [36]. This trend was not observed in either study done by us or by Kamath et al. [18]. The anticipated higher average break stress at low humidity was reduced by the significant presence of prematurely broken fibres (broke at low stresses). Meanwhile, the average break stress at high humidity did not decrease proportionally as observed in Caucasian hair [36] because less textured hair fibres broke prematurely. This compensated for the anticipated lower average break stress at high humidity. Thus, average break stresses of textured hair at high and low humidities are not different.

Although dimensional data suggest significant penetration by low‐MW compounds, it appears that they did not have any protein stabilizing effect due to their small molecular size. Meanwhile, both mid‐ and high‐MW peptides were effective at improving breakage performance of relaxed hair. As fluorescence work revealed different depth of actives penetration, we hypothesize that the reduction in breakage for mid‐ and high‐MW peptide treated hair may be due to different mechanisms. Mid‐MW peptides, comprised of ~20 amino acids, were made using a mild proprietary process that leaves the natural cystine content of the keratin in an active, S‐sulpho form. Besides potentially stabilizing the hair from the inside by creating salt‐linkages, this peptide may also have created covalent bonds to some extent via ‐S‐S‐ ‐SH interchange at several locations inside the hair. Small increases in denaturation temperature in comparison with the control supports this proposed mechanism of action. Literature also agrees that peptides containing disulphide bonds have a high affinity to hair keratins [14].

High‐MW peptides, because of size restriction, could not penetrate deeper than the cuticle layer or cracked cortex. Alternatively, high‐MW peptides can unfold and adsorb on the surface forming a cohesive film and filling the cracks. The film was visible at 10 000× images, and the peptides were not rinsed from the cracks as effectively as from the surface. We postulate that the strength of the thin proteinaceous film was able to suppress the premature breakage (reduced crack formation and delayed the crack propagation) resulting in higher break stresses than if fibre would have broken prematurely. This was indirectly confirmed by the data presented in Table 1 (lower amount of premature breakage observed). To confirm or disprove these hypotheses, additional experiments must be designed and executed.

Our findings showing partial hair strength recovery after treatment with mid‐ and high‐MW peptides are in agreement with the trends observed by research groups who studied (i) mid‐MW peptide effect on relaxed African hair [34], (ii) mid‐MW peptide effect on bleached Caucasian hair [15] and (iii) high‐MW protein effect on over‐bleached Caucasian hair [35]. Similar trends of mid‐MW peptide effects on bleached hair tensile strength were observed by Barba et all; however, the significance of these results was compromised by the sample size being very small (*N* = 10) [10].

## Conclusions

To the best of our knowledge, ours is the first study that compares the effect of low‐, mid‐ and high‐MW peptides on relaxed hair using the same test conditions. The results from this study show that the high‐MW keratin peptides only penetrated into the surface layers of relaxed textured hair, whereas the mid‐range keratin peptides were able to penetrate deeper into the cortex. However, both the mid‐range and high‐MW keratin peptides were able to improve breakage parameters: increase in break stress and reduce the number of premature fractures. Although fibre dimension measurements suggest significant penetration of the low‐MW keratin peptides and leucine (significantly increased cross‐sectional area of the hair), it appears that these compounds did not have any protein stabilizing effect. Our data, therefore, suggest that low‐MW compounds may have a volume increase effect, and mid‐ and high‐MW peptides may indeed have a damage repair effect on freshly relaxed textured hair.

## Conflicts of interest

The authors report no conflicts of interest.
